# Research on Multimodal Control Method for Prosthetic Hands Based on Visuo-Tactile and Arm Motion Measurement

**DOI:** 10.3390/biomimetics9120775

**Published:** 2024-12-19

**Authors:** Jianwei Cui, Bingyan Yan

**Affiliations:** Institute of Instrument Science and Engineering, Southeast University, Nanjing 210096, China; 220213676@seu.edu.cn

**Keywords:** intention recognition, human–machine interaction, 2D Lidar, environmental perception, prosthetic hand

## Abstract

The realization of hand function reengineering using a manipulator is a research hotspot in the field of robotics. In this paper, we propose a multimodal perception and control method for a robotic hand to assist the disabled. The movement of the human hand can be divided into two parts: the coordination of the posture of the fingers, and the coordination of the timing of grasping and releasing objects. Therefore, we first used a pinhole camera to construct a visual device suitable for finger mounting, and preclassified the shape of the object based on YOLOv8; then, a filtering process using multi-frame synthesized point cloud data from miniature 2D Lidar, and DBSCAN algorithm clustering objects and the DTW algorithm, was proposed to further identify the cross-sectional shape and size of the grasped part of the object and realize control of the robot’s grasping gesture; finally, a multimodal perception and control method for prosthetic hands was proposed. To control the grasping attitude, a fusion algorithm based on information of upper limb motion state, hand position, and lesser toe haptics was proposed to realize control of the robotic grasping process with a human in the ring. The device designed in this paper does not contact the human skin, does not produce discomfort, and the completion rate of the grasping process experiment reached 91.63%, which indicates that the proposed control method has feasibility and applicability.

## 1. Introduction

The prosthetic hand is an important tool to restore hand function for people with upper limb disabilities. Accurate recognition of the intention of upper limb actions is essential for controlling coordinated actions of a prosthetic hand. The coordinated actions of the human hand can be summarized in two parts: postural coordination and process coordination. The information that describes upper limb action patterns and intentions includes bioelectric, bionic, and kinematic signals [1].

Bioelectric signals are mainly composed of electroencephalography (EEG) and electromyography (EMG) signals. EEG signals can directly reflect intention information in the human brain about 100 to 150 milliseconds ahead of limb actions [2], which is suitable for the intention recognition and control of robotic arms and prosthetic hands. Baoguo Xu et al. studied EEG signals of humans during left, right, up, and down actions and successfully controlled the action of a robot and grasped the target object [3]. Kiyoshi Nagai et al. implemented EEG information to trigger robot actions [4]. However, EEG signals are usually very weak [5,6] and are affected by environmental noise. Hence, intention recognition using EEG signals has poor accuracy and stability. In addition, only simple action control can be accomplished.

Note that changes in electrical signals, EMG signals, can be measured on the surface of the skin when human muscles move a limb. These electrical signals can rapidly reflect limb action intentions and can be applied in the field of prosthetic hands. By using EMG signals, researchers have investigated strategies to control a pneumatic robotic arm to complete grasping actions [7], as well as methods to control a prosthetic hand to complete actions such as closing, opening, wrist flexion, and double wrist flexion using single-channel surface EMG signals [8]. Reference [9] proposed a new human–computer interaction for tetraplegic patients, which was evaluated using EMG in five patients with spinal cord injuries. The preliminary results provided a new solution for advanced spinal cord injury patients. In addition, EMG signals can also be used to control a prosthetic hand to perform certain fine actions, such as axial hole assembly [10]. Based on EMG and eye action signals, Keisuke Shima et al. controlled a prosthetic hand to help a disabled person eat [11]. Segil et al. fitted four EMG electrodes to the missing forearm region to control a prosthetic hand, achieving an average of 55% physiological function in patients with limb loss [12].

Previous studies on prosthetic hands have indicated that EMG signals are superior to EEG signals. However, there are many problems with EMG signals. Although reference [12] provided examples of electrodes attached to the amputation site of an arm, the muscles of the arm of a disabled individual often show varying degrees of atrophy. Muscle atrophy leads to weaker and noisier EMG signals, making it difficult to acquire and process them. Many previous works did not consider how to coordinate the actuation of multiple muscles, which not only makes the acquisition of EMG signals difficult but also makes them time-consuming and difficult to use. In addition, EMG electrodes can cause skin irritation and serious malfunctions due to interference from factors such as humidity.

It is worth noting that body actions are the result of the human brain’s intentions, i.e., they reflect human intentions. Body motion information includes velocity, acceleration, posture, position, etc. This body motion information can be measured using inertial sensors. With the development of MEMS, inertial sensor technology has matured [13] and has become a commonly used sensor to collect human motion parameters, gradually becoming applied in the field of prosthetic control. For example, Ben et al. introduced an inertial measurement unit (IMU)-based method to recognize the action intention of human lower limbs [14]. Samuel et al. illustrated the drawbacks of EMG signals and proposed an algorithm for human gesture recognition integrating an IMU and EEG signals [15]. Noccaro et al. recorded arm actions for human-like upper limb actions using a robotic arm based on an IMU [16]. Merad et al. designed a coordinated elbow motion prediction method based on IMU upper limb kinematics, which provided ideas for flexible control of prosthetic limbs [17]. The prosthetic hand research team at Southeast University investigated the use of inertial sensors to control shoelace tying with prosthetic hands, with good results [18].

In recent years, multimodal signals such as visual, haptic, and speech signals have been widely used for prosthetic limb control to achieve precise manipulation of prosthetic limbs. Martin Harold et al. designed an upper limb prosthesis control method integrating visual, inertial, and EMG signals [19]. Ghazaei et al. proposed a control method based on the fusion of visual and EMG information, which successfully realized the task of grasping 500 different types of objects [20]. It can be seen that visual information is important for the control of a prosthetic hand, but a prosthetic hand is only suitable for monocular vision, and it is difficult to accurately obtain the dimensions of objects due to a lack of depth information, coupled with the facts that there are many different types of objects and the dimensions of the same type of object can also be very different, which makes monocular-vision-based control methods cumbersome and difficult to adapt to complex environments and tasks. In order to solve this problem, the use of Lidar as a visual perception sensor, which can provide rich depth information, can effectively compensate for the shortcomings of monocular vision and is able to efficiently acquire features such as object dimensions [21]. There have also been studies on controlling a prosthetic hand with voice signals, which had better results in quiet situations, but this voice control method can often be embarrassing for users.

Therefore, in this paper, we propose a multimodal control algorithm for prosthetic hands based on opto-haptic and arm kinematic measurements, which is used to solve the cross-sectional shape and size recognition and dynamic gesture adjustment of target objects in complex scenes. The main contributions can be categorized into the following three aspects:

(1) In this paper, a new type of prosthetic hand control system integrating visual and tactile sensing and arm motion measurement is constructed, and a cross-sectional object shape and size recognition method based on the fusion of a monocular camera and two-dimensional Lidar is proposed in a pioneering manner. At the same time, a dynamic grasping control algorithm with multimodal information fusion is designed, which combines the upper limb motion state and lesser toe tactile information to realize intelligent recognition and dynamic gesture adjustment for objects in the middle of the target area in a complex environment, providing a brand-new technological path for the intelligent control of prosthetic hands.

(2) In this study, an algorithm for recognizing the cross-sectional shape and size of objects based on the fusion of a monocular camera and 2D Lidar data is proposed for the first time. The algorithm recognizes and classifies the shape of an object through the YOLOv8 model and extracts geometric features, and then combines 2D Lidar with the DBSCAN algorithm to cluster the objects, with the DTW algorithm used to further recognize the cross-sectional shape and size of the grasped part of the object, realizing grasping attitude control of a prosthetic hand. The method in this paper overcomes the limitations of a single sensor in complex environments, significantly improves the accuracy and robustness of object recognition, and reduces costs at the same time.

(3) In this study, a grasping action control algorithm based on the fusion of an IMU and haptic sensor data is proposed. The upper limb motion state is identified using a sliding window attitude angle variance calculation algorithm, a palm motion model is established, and the palm position is classified using the MLP (multi-layer perceptron) algorithm; the foot haptic data are processed using Kalman filtering, and the pressing action of the lesser toes is identified by combining with the peak extraction algorithm. By integrating the upper limb kinematic state, hand position, and foot haptic information, precise optimization of the grasping action is achieved. Compared with traditional EMG, EEG, or voice control methods, this algorithm enhances the flexibility, covertness, and comfort of operation through natural human interaction.

The rest of this paper is organized as follows: in Section 2, we present the overall design of the prosthetic hand control system; Section 3 introduces an algorithm for recognizing the shape and size of an object cross-section based on the fusion of a monocular camera and 2D Lidar data; Section 4 details a grasping action control algorithm based on the fusion of an IMU and haptic sensor data; Section 5 is the experimental part, which confirmed the feasibility of the methodology proposed in this paper through experiments, and Section 6 provides the conclusions and future perspectives.

## 2. System Design

**Figure 1:**
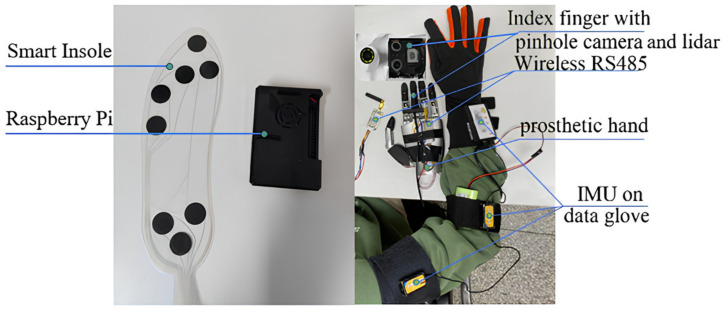
Hardware composition of the prosthetic hand control system.

The overall structure of the prosthetic hand control system is shown in Figure 1 and consists of the main control module (Raspberry Pi), Lidar, pinhole camera, IMU, flexible pressure sensing insole, and prosthetic hand. Among them, the pinhole camera has a size of φ3.9 × 20 mm and the laser of the Lidar has a laser size of 6 × 6 × 8 mm, which can be integrated and mounted on the finger.

**Figure 2:**
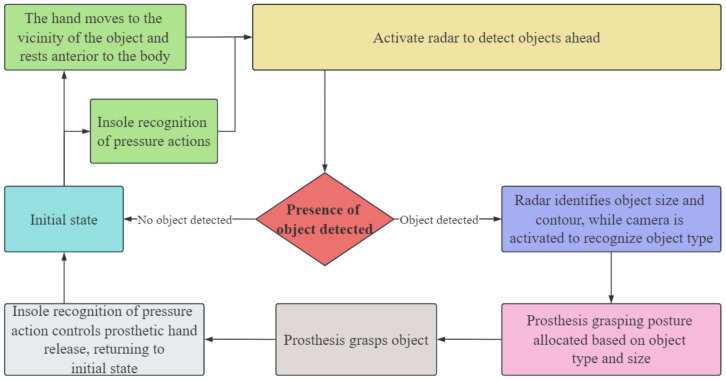
Prosthetic hand control system flow.

The grasping action of the human hand is a complex process that requires the synergistic cooperation of multiple organs and is the result of the fusion processing of human intention and environmental perception. Based on an analysis of the complete grasping action process, this paper designed a complete control system for the prosthetic hand action process, as shown in Figure 2. In this system, IMUs are worn on the large and small arms to detect the movement of the upper limbs in real time and determine the position of the end of the hand, the Lidar and camera are mounted on the prosthetic hand for recognizing the size and type of the object, and flexible pressure sensing insoles are placed on the soles of the feet to detect the user’s active manipulation signals. The design concepts of this system include the following: first, an IMU is used to obtain the velocity information of the upper limb to judge its motion state, and a D-H (Denavit–Hartenberg) model of the upper limb is established to solve the position of the hand relative to the body, so as to judge the grasping timing; second, unlike a traditional prosthetic hand system that recognizes the specific class of an object, this paper uses camera vision to recognize the shape of the object; third, this paper innovates with the use of 2D Lidar to recognize the object size, designing point cloud preprocessing and size calculation algorithms; fourth, the foot pressing action is used as a human–computer interaction signal; fifth, a grasping strategy for the prosthetic hand based on the shape and size of the object is designed, and the above information is fused to control the action process of the prosthetic hand.

The control process of the prosthetic hand designed in this system is as follows: The initial state of the system is that the upper limbs are static and naturally drooping, when it is necessary to grasp an object, the arm drives the robotic hand to move towards the object. When the IMU detects that the upper limbs have stopped moving and the hand is located in front of the body, this means that the robotic hand can reach the vicinity of the object. At this time, the Lidar is used to detect whether there is an object in front of the body, if no object exists, this means that no movement of the prosthetic hand is needed, if there is an object, the camera acquires an environment image, and together with the Lidar, it recognizes the type and size of the object, and then the system controls the prosthetic hand to adjust the posture for grasping. When the object needs to be released, the flexible pressure sensing insole recognizes the user’s foot pressing action, allowing the prosthetic hand to release the object and return to the initial state.

## 3. Monocular Camera and 2D Lidar-Based Object Cross-Section Shape and Size Identification Method

### 3.1. Overview of the Methodology

Monocular cameras are small, lightweight, and low-cost, but a lack of depth information makes it difficult to accurately obtain the size of an object, coupled with the fact that there are many types of objects and the size of the same type of object can vary greatly, which makes monocular-vision-based control methods cumbersome and difficult to adapt to complex environments and tasks.

Lidar is commonly used for slam navigation in mobile robots. Lidar illuminates the target in the sector plane and can obtain a distance value corresponding to the emission angle, and point cloud data are formed through multiple measurements. The point cloud data of Lidar are suitable for long-distance measurement and are often used to describe the traveling path of mobile robots. In this paper, the point cloud data of Lidar were used to measure the cross-sectional shape and size of the grasped object, and the main problems that needed to be solved were the small size of the measurement target, the short measurement time, the small amount of measurement data obtained, and the large error. In addition, the random jitter of a human hand increased the measurement error and difficulty.

In order to solve the above problems, a monocular camera was first used to recognize and classify the shape of the object using the YOLOv8 model to extract geometric features, and then the DBSCAN algorithm was utilized in combination with 2D Lidar to cluster the objects, with the DTW algorithm used to further identify the cross-sectional shape and size of the object’s grasped part, in order to realize control of the grasping posture of the prosthetic hand.

### 3.2. Monocular Camera Object Shape Recognition Method

**Figure 3:**
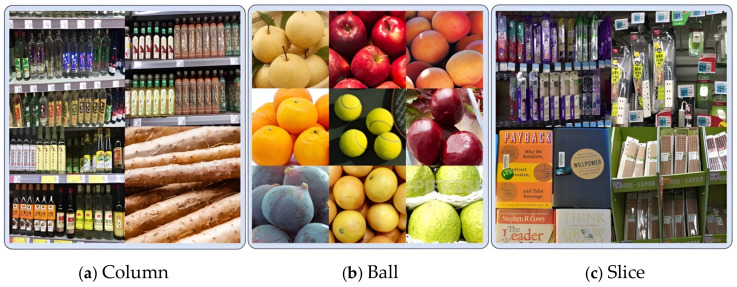
Dataset classification.

Most camera vision algorithms for prosthetic hands choose to recognize specific classes of objects. However, there are many types of objects and it is difficult to cover them comprehensively. Including more objects will lead to a wide range of classes, affecting the recognition accuracy and efficiency. Based on the above analysis and the fact that common relatively regular objects have relatively simple shapes, and different objects of the same shape have a great similarity in their corresponding grasping gestures, this section divides common objects into three categories, i.e., columns, balls, and slices, from the point of view of the design of the grasping gesture. YOLO is a widely used and validated target detection and recognition model. In this section, the shape of the object is recognized using YOLOv8 [22]. The images used in this method were sourced from Coco, supermarket, and fruit scenes, etc., and were labeled to form a new dataset. Figure 3 shows some of the objects in each category in the dataset. The dataset generated in this section consists of a total of about 21,000 images, including 54 types of columns, 113 types of balls, and 16 types of slices, and the dataset is divided into training, validation, and testing sets in a ratio of 8:1:1.

**Figure 4:**
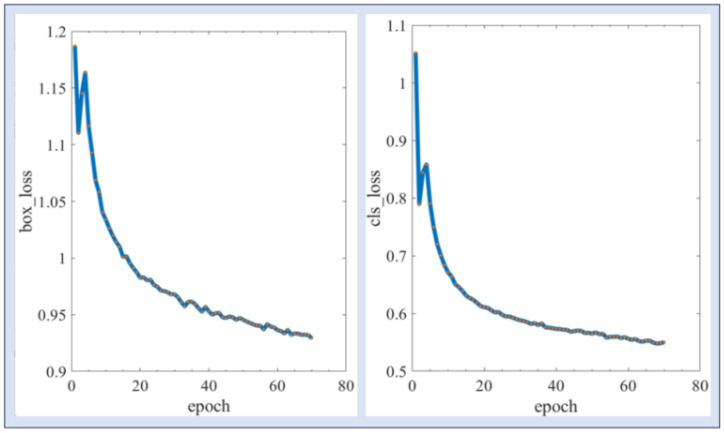
Training loss curve.

**Figure 5:**
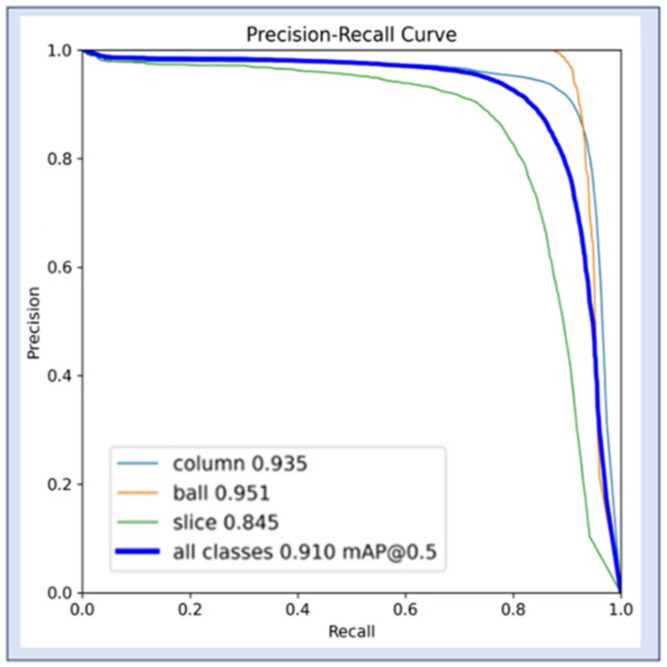
mAP curve.

The training in this paper was caried out on the Ubuntu 20.4 + PyTorch platform, the hardware configuration of the machine is shown in Table 1, and the initial model chosen for training was YOLOv8. The loss curve of the training model is shown in Figure 4, box_loss represents the loss in recognizing the box, cls_loss represents the loss in classifying, the horizontal axis represents the number of iterations, and the vertical axis represents the value of the loss. It can be seen that the loss decreased faster in the first 20 rounds and slowly decreased later. mAP is an important metric for evaluating tasks such as target detection and segmentation in computer vision, and it is well suited for evaluating the accuracy of a model, which is the average of the AP (average value of precision). The AP responds to the area enclosed by the precision–recall curve and the horizontal and vertical axes, and the mAP is computed as shown in Equation (1),

(1) $$mAP=\frac{\sum_{i=1}^{n}\frac{\sum_{k=1}^{R_{i}}P\left(k\right)∆Re\left(k\right)}{R_{i}}}{n}$$

where n is the number of categories in the detection task, $R_{i}$ is the total number of true positive cases for category i, $P\left(k\right)$ is the precision of category i in the first k detections, and ∆Re(k) is the difference in recall when category *i* changes from i−1 to i. Figure 5 demonstrates the mAP variation curve during the training process of this training set, and it can be seen that the mAP value reached 0.91, which represents good performance for the model.

### 3.3. Pre-Processing of Lidar Point Cloud

Firstly, the point cloud data needed to be filtered and put through a noise reduction process. Because the research object of this paper was a prosthetic hand, we could only use a small size of Lidar, the data density was low but the frame rate was high, and the point cloud data were in sparse matrix form. The slam algorithm point cloud filtering method commonly used in mobile robots is not ideal for a more sparse point cloud. In this paper, we proposed a multi-frame noise reduction algorithm for point cloud filtering by referring to the multi-frame noise reduction algorithm in image processing. This algorithm is utilized by continuously irradiating the same cross-section of an object in a split second to obtain multiple frames of a two-dimensional point cloud, and then the coordinates of the same position are averaged to obtain a set of two-dimensional point cloud data after noise reduction. The calculation formula is shown in Equation (2), where N is the number of frames, which can be obtained as a set of points $P_{i}\left(x_{i},y_{i}\right),i=1,2,3,\ldots$.

(2) $$\left\{\begin{array}{c} x_{i}=\frac{1}{N}\sum_{j=1}^{N}x_{ij}\\ y_{i}=\frac{1}{N}\sum_{j=1}^{N}y_{ij} \end{array}\right.$$

**Figure 6:**
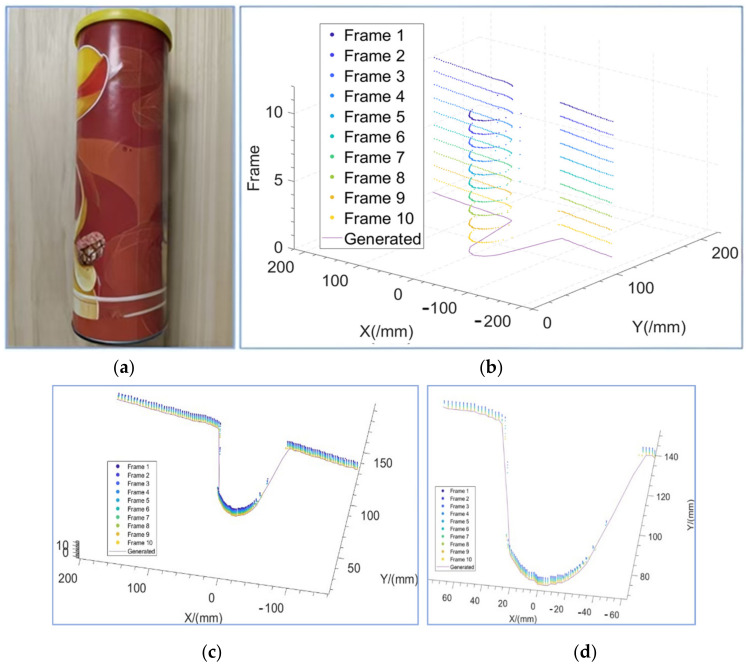
Noise reduction algorithm for object point cloud. (**a**) Environment. (**b**) Continuous multi-frame point cloud. (**c**) Continuous multi-frame point cloud overlay. (**d**) Continuous multi-frame point cloud overlay localized to the object.

Figure 6a shows a cylindrical object and the Lidar scans of the object. A multi-frame 2D point cloud of a certain cross-section was obtained, as shown in Figure 6b. The multi-frame point cloud in Figure 6b was processed using the above multi-frame noise reduction algorithm to obtain the result in Figure 6c. The processed points were connected to obtain the solid line in Figure 6c. The local area of the cylindrical object is zoomed in as shown in Figure 6d. The multi-frame point cloud is distributed close to both sides of the solid line, which indicates that the multi-frame noise reduction algorithm played a filtering role in the above error.

The Lidar used in the system emits a laser beam from the same point, similarly to a point light source. In addition, different objects illuminated by the Lidar are separated on the 2D point cloud map, and the same object forms a set of neighboring points. Therefore, this section used the DBSCAN (density-based spatial clustering of applications with noise) clustering algorithm to extract the objects and a method to also filter the points that were separated from the object caused by random errors, i.e., isolated point exclusion method. DBSCAN is a density-based clustering algorithm that clusters points in dense regions, while marking points in sparse areas as noise. The core idea of DBSCAN is to expand clusters from core points, which are points that have a sufficient number of neighboring points within a certain distance. The two main parameters of the algorithm are eps and min_samples, where eps represents the maximum distance between two points to define the neighborhood, while min_samples represents the minimum number of neighboring points required to form a cluster.

**Figure 7:**
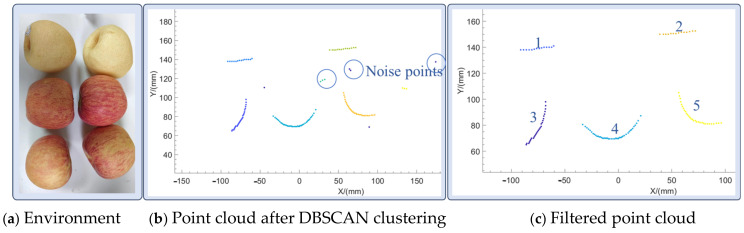
Multiple object DBSCAN filter.

Figure 7 demonstrates the process of filtering a fruit pile using the DBSCAN clustering algorithm. When the environment in Figure 7a was scanned using Lidar, a two-dimensional point cloud map of the cross-section could be obtained. Clustering this point cloud using the DBSCAN algorithm led to the result of Figure 7b, which contains multiple noise points, e.g., at the place circled by the circle. After the removal of the isolated noise points, the remaining point cloud consisted of two parts, i.e., the real object point cloud and the clustered noise point cloud, to filter out the clustered noise as much as possible. Each cluster of the point cloud was processed by using the curve fitting method, considering that the method in this chapter aims to identify relatively regular objects and noise is usually irregular. Moreover, these characteristics were reflected in the curve fitting polynomials, which were within the error tolerance. True objects have lower powers and noise has higher powers. After the above processing, Figure 7c yields the real objects, numbered 1 and 2 for the tabletop and numbered 3–5 for the spherical fruit.

As seen in Figure 7c, a complex environment containing multiple objects can be processed to obtain multiple real objects. However, the hand only wants to grasp one of them, and because the hand will be close to the object to be grasped, the real object to be grasped is the one that is close to the origin, such as object No. 4 in Figure 7c.

### 3.4. Recognizing the Shape and Size of the Object’s Cross-Section

After processing in the above subsections, the method in this research locates the target object. In this section, we identify the shape and size of the object cross-section. The identification of the cross-section shape is used to obtain the size of the object, because the sizing algorithm is different for different shapes of the cross-section. According to the common object shapes, this method divides the cross-section shapes into three categories, i.e., circular, straight-line segment, and L-shaped. The dimensions of this method were used to design the grasping posture of the prosthetic hand. Therefore, the dimensions are the effective grasping dimensions, i.e., for the circular cross-section, the dimension is the diameter, for the straight-line segment cross-section, the dimension is the straight-line segment length, and for the L-shape cross-section, the dimension is the length of the first and last point connecting lines.

**Figure 8:**
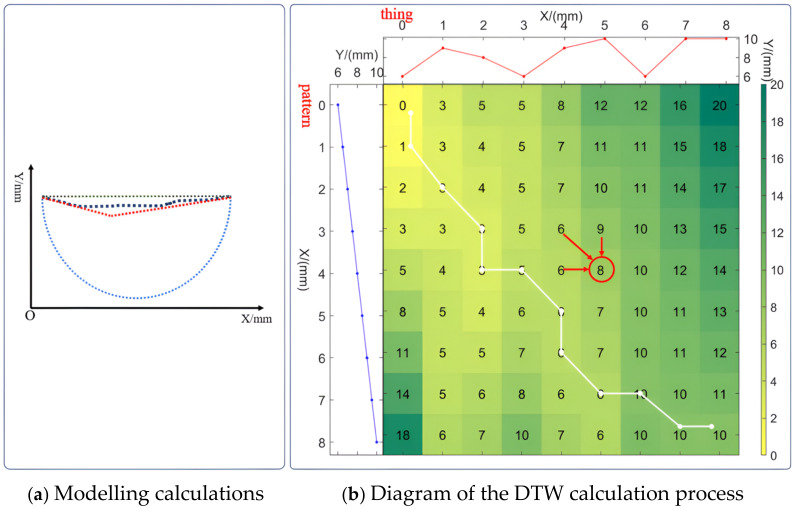
Algorithm for point cloud DTW similarity calculation.

The classification problem of cross-sectional shapes can be transformed into a similarity problem with respect to semicircles, lines, and L-shaped lines. The semicircles, lines, and L-shaped lines are generated based on the cross-section to be identified, which is referred to as the template in this paper, and the specific generation method is shown in Figure 8a. The black dotted line represents the 2D point cloud of the object’s cross-section scanned by the Lidar system. The blue dotted line represents the generated semicircular point cloud, the red dotted line represents the L-shaped linear point cloud, and the green dotted line represents the linear point cloud. The 2D point cloud can be decomposed into an x-direction sequence $\left\{x_{1},x_{2},...,x_{n}\right\}$ and y-direction sequence $\left\{y_{1},y_{2},y_{3},...,y_{n}\right\}$, and the template point cloud is generated as shown in Equation (3).

(3) $$\left\{\begin{array}{c} y_{i}^{s}=\frac{y_{n}-y_{1}}{x_{n}-x_{1}}\left(x_{i}-x_{1}\right)+y_{1}\\ y_{i}^{c}=\frac{y_{1}+y_{n}}{2}-\sqrt{\frac{{\left(x_{n}-x_{1}\right)}^{2}+{\left(y_{n}-y_{1}\right)}^{2}}{4}-{\left(x_{i}-\frac{x_{1}+x_{n}}{2}\right)}^{2}}\\ y_{i}^{l}=\frac{y_{2}-y_{1}}{x_{2}-x_{1}}\left(x_{i}-x_{1}\right)+y_{1}\;,\;x_{i}\leq \frac{y_{n}-y_{1}+\frac{y_{2}-y_{1}}{x_{2}-x_{1}}x_{1}-\frac{y_{n}-y_{n-1}}{x_{n}-x_{n-1}}x_{n}}{\frac{y_{2}-y_{1}}{x_{2}-x_{1}}-\frac{y_{n}-y_{n-1}}{x_{n}-x_{n-1}}}\\ y_{i}^{l}=\frac{y_{n}-y_{n-1}}{x_{n}-x_{n-1}}\left(x_{i}-x_{n}\right)+y_{n},x_{i}>\frac{y_{n}-y_{1}+\frac{y_{2}-y_{1}}{x_{2}-x_{1}}x_{1}-\frac{y_{n}-y_{n-1}}{x_{n}-x_{n-1}}x_{n}}{\frac{y_{2}-y_{1}}{x_{2}-x_{1}}-\frac{y_{n}-y_{n-1}}{x_{n}-x_{n-1}}} \end{array}\right.$$

where $y_{i}^{s},y_{i}^{c},y_{i}^{l}$ are straight-line, circular, and L-shaped point cloud templates, respectively. In this section, a dynamic time warping algorithm (DTW) is used to classify the cross-section shapes. The DTW algorithm is used to compare the similarity of two sequences, and it is able to compute the distance between the two sequences. The process of using the DTW algorithm to compute the cross-section point cloud sequences and template point cloud sequences is shown in Figure 8b, with the top part of the object’s cross-section point cloud and the left side of the template point cloud of the generated straight-line segments. In the middle part of the process of distance calculation for the two sequences, as shown in Equation (4), $D_{ij}$ is the cumulative distance from $pattern_{i}$ to $thing_{j}$, as shown in the red circle in Figure 8b, $D_{ij}$ is obtained using the sum of the three worthwhile minimums in the upper left corner and the distance from the current position, and the cumulative distance between any two points of the template and the object point cloud can be obtained after traversal. Let n be the number of points in the object and template point clouds, and $D_{\left\{nn\right\}}$ be the shortest distance between two points from the current template and the object’s cross-section. In Figure 8b, the white broken line represents the path that achieves the shortest distance. By calculating the distances between the points in the object’s cross-section point cloud and the sequences of the three template point clouds, the template corresponding to the shortest distance determines the classification of the object’s cross-section.

(4) $$\left\{\begin{array}{l} D_{ij}=\min\left(D_{i-1,j},D_{i-1,j-1},D_{i,j-1}\right)+\left|thing_{j}-pattern_{i}\right|,i,j=0,1,\ldots \\ D_{-1,j}=\infty ,\;D_{i,-1}=\infty \end{array}\right.$$

Using the above algorithm, the shape of the cross-section of the object to be recognized by the Lidar can be obtained in this section, and the size of the cross-section can be easily calculated based on the shape. If the object is a straight segment type or L-shape, its cross-section dimensions are presented in Equation (5), as follows:

(5) $$size=\sqrt{{\left(x_{n}-x_{1}\right)}^{2}+{\left(y_{n}-y_{1}\right)}^{2}}$$

where $\left(x_{1},y_{1}\right)$ is the start point of the cross-section point cloud of the object, $\left(x_{n},y_{n}\right)$ is the end point of the cross-section point cloud of the object, and size is the requested size. If the object is a circular, the steps for sizing its cross-section are as follows: first, the Equation of the circle is fitted using the least squares circular fitting method, and then its diameter is the requested size. The least squares fitting point cloud circular fitting method is as follows: first, determine the parameters of the circle to be fitted, i.e., ${\left(x-a\right)}^{2}+{\left(y-b\right)}^{2}=r^{2}$, where (a,b) is the center of the circle and r is the radius, and the error sum is calculated as shown in Equation (6), where N is the number of points,$\left(x_{i},y_{i}\right)$ are the coordinates of the cloud of points, and Q(a,b,r) is the error sum of the cloud of points, so that the set of error sums which are minimum (a,b,r) is the parameter of the fitted circle. And the parameters can be obtained by taking the partial derivatives of (a,b,r) and assigning a value of 0.

(6) $$\left\{\begin{array}{l} d_{i}^{2}={\left(x_{i}-a\right)}^{2}+{\left(y_{i}-b\right)}^{2}\\ \delta_{i}=d_{i}^{2}-r^{2}\\ Q\left(a,b,r\right)=\sum_{i=1}^{N}\delta_{i}^{2}=\sum_{i=1}^{N}{\left(x_{i}^{2}+y_{i}^{2}-2ax_{i}-2by_{i}+a^{2}+b^{2}-r^{2}\right)}^{2}\;\\ \frac{\partial Q}{\partial a}=0,\frac{\partial Q}{\partial b}=0,\frac{\partial Q}{\partial r}=0 \end{array}\right.$$

## 4. Multi-Sensor-Based Control Method for Prosthetic Hand

### 4.1. IMU Recognizes Upper Limb Motion State and Hand Position

According to the analysis in Section 2, a prosthetic hand system needs to recognize the motion state of the upper limb, including the motion and static state, and the motion state of the upper limb is determined by the velocity, which can be represented by the difference in the attitude angle. The built-in accelerometer of the IMU can capture data of the roll angle, the yaw angle, and the pitch angle. Hence, wearing the IMU on the upper limb can determine the motion state of the upper limb. In this section, the difference in attitude angle is calculated by the following process:

(1) A sliding window is used to calculate the moving variance of the attitude angles acquired by the IMU, with a sampling rate of 50 Hz and a window length l=10, i.e., 10 sampling points.

(2) Calculate the variance of the three-axis attitude angles of the current window as shown in Equation (7), where $\emptyset_{k-i}$, $\theta_{k-i}$, $\varphi_{k-i}$ are the values of the turn angle, yaw angle, and pitch angle of the l−i+1st point of the current window, respectively, and $\bar{\emptyset },\bar{\theta },\bar{\varphi }$ is the mean value of the attitude angles in the current window.

(7) $${Var}_{k}=\left\{\begin{array}{c} 0\;\;\;\;\;\;\;\;\;\;\;\;\;\;\;\;\;\;\;\;\;\;\;\;\;\;\;\;\;\;\;\;\;\;\;\;\;\;\;\;\;\;\;\;\;\;\;\;\;\;\;\;\;\;\;\;\;\;\;\;\;\;\;\;\;\;\;\;\;\;\;\;\;\;\;\;\;\;\;\;\;,\;k\leq l\\ \frac{1}{l}\sum_{j=1}^{l}{[\left(\emptyset_{k+i}-\bar{\emptyset }\right)}^{2}+{\left(\theta_{k+i}-\bar{\theta }\right)}^{2}+{\left(\varphi_{k+i}-\bar{\varphi }\right)}^{2}],\;\;k>l \end{array}\right.$$

**Figure 9:**
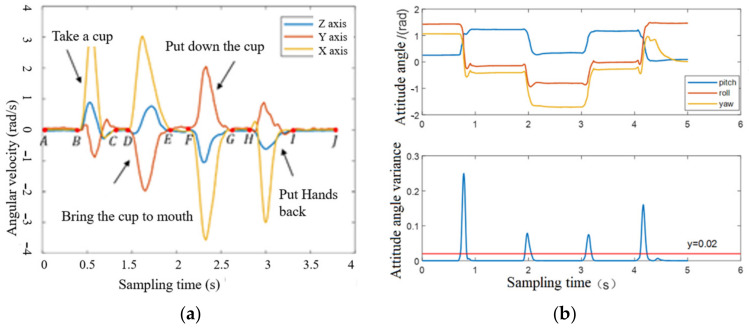
(**a**) Acceleration changes while drinking water. (**b**) Attitude angle changes while wearing glasses.

Figure 9a shows the acceleration change during the process of drinking water. The change in angular velocity indicates that there is movement in the upper limb. In the process of picking up the cup, i.e., moving the cup to the mouth, putting down the cup, and putting down the hand, the acceleration and posture angle change obviously. The acceleration stays near 0 rad/s and the posture angle stays stable for the rest of the time. The movement variance change curve of putting on glasses is shown in Figure 9b, and the curve shows obvious wave peaks when the arm is in a motion state. Therefore, this can be considered as a stationary state by setting the threshold th, when $Var_{k}\leq th$, otherwise it is considered as a motion state. During the rest of the time, the variance value is close to 0. According to the analysis of the angular velocity curves of the above actions, it can be seen that at the moment of grasping or opening the palm, the upper limb is in a stationary state, and the angular velocity of the upper limb is close to 0 rad/s (e.g., segments $C_{1}D_{1},E_{1}F_{1}$ and $G_{1}H_{1}$). Hence, the upper limb will only change the grasping and releasing state when it is at rest, which is the first rule of the upper limb motion state transition, i.e., the dynamic static rule.

**Figure 10:**
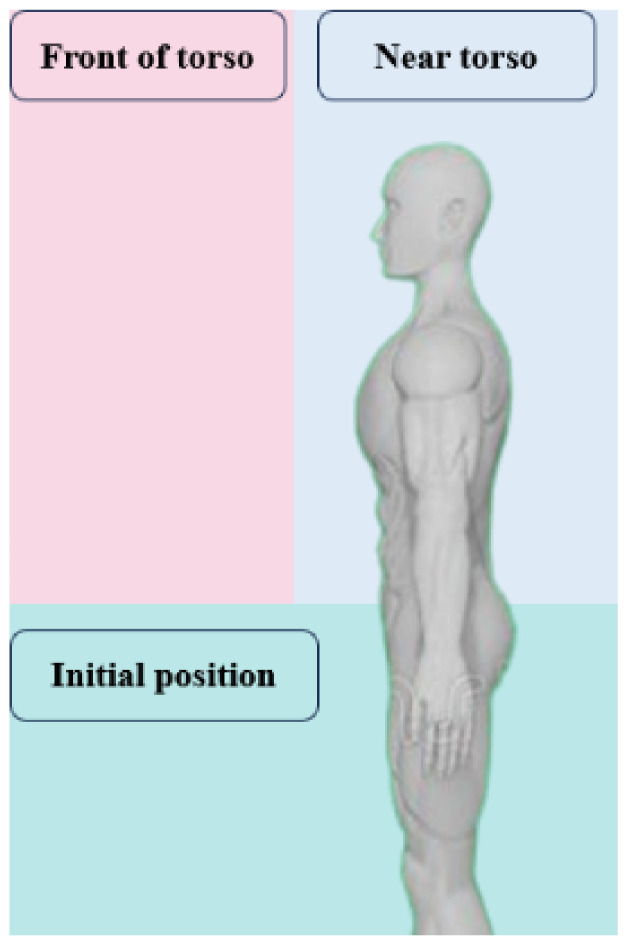
Division of the end position of the hand.

**Figure 11:**
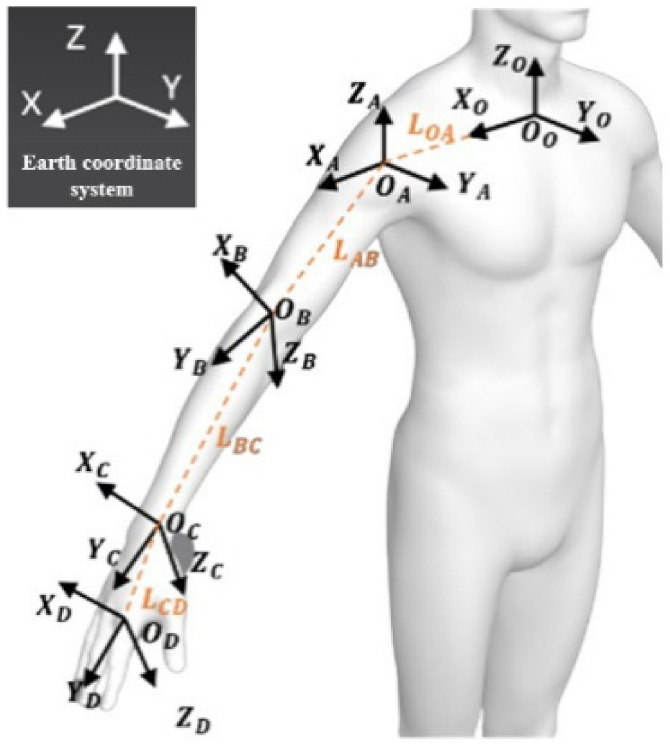
D-H model of the upper limb.

According to the analysis in Section 2, the user acquires the end position of the hand when using the prosthetic hand to complete an action and divides the end position of the hand into the initial position, in front of the torso, and the position near the torso, as shown in Figure 10. To further analyze the end position of the hand, a D-H model of the upper limb was established for positive kinematic analysis in this subsection, as shown in Figure 11, with points O, A, B, C, and D representing the chest, shoulder, elbow, wrist, and palm positions of the human body, respectively. $O_{O}-X_{O}Y_{O}Z_{O}$,$O_{A}-X_{A}Y_{A}Z_{A}$,$O_{B}-X_{B}Y_{B}Z_{B}$,$O_{C}-X_{C}Y_{C}Z_{C}$,$O_{C}-X_{D}Y_{D}Z_{D}$ denote the base coordinate system of the corresponding position, respectively. And $L_{OA},L_{AB},L_{BC},L_{CD}$ denote the dimensions of the arm, i.e., half shoulder width, upper arm length, forearm length, and palm length. The D-H equation of the hand is shown in Equation (8), and the coordinates of the hand in the chest coordinate system are obtained by multiplying the four transformation matrices with the coordinates of the palm coordinate system. After the above positive kinematic analysis, the coordinates of the end of the hand could be obtained. To classify the position of the end of the hand, a multilayer perceptual machine model was introduced in this section. The *x*, *y*, *z*-axis coordinate values of the end of the hand, $P_{D}^{x},P_{D}^{y},P_{D}^{z}$, and the angles between the three-axis attitude angles, ∆∅,∆θ, and ∆φ, for the large and small arms were selected as the eigenvalues. The MLP had six input neurons and three output neurons. The input to the MLP consisted of $P_{D}^{x},P_{D}^{y},P_{D}^{z}$ and ∆∅,∆θ,∆φ, while the output was the position of the hand, representing the front-of-torso, near-torso, and initial positions. The training data were collected from input data obtained from different volunteers. The training dataset had nearly 5000 sets of data. The parameters of this model were chosen to have two hidden layers with 10 and 6 neurons, respectively, and the learning rate was 0.1. The accuracy of the classification of the 100 sets of data using the trained model was 98%.

(8) $$\left[\begin{array}{c} P_{O}^{D}\\ 1 \end{array}\right]=T_{A}^{O}T_{B}^{A}T_{C}^{B}T_{D}^{C}\left[\begin{array}{c} P_{D}^{D}\\ 1 \end{array}\right]$$

### 4.2. Foot Pressing Action Recognition

Research has shown that there are two states of the human foot during daily activities: the static state (e.g., sitting, standing, etc.) and the locomotion state (e.g., walking, running, etc.). In the above states, foot strength is mainly concentrated in the forefoot and heel, but increasing the tactile force in these two areas is very difficult and may also affect balance, making them unsuitable for characterization signals. In contrast, the lesser toe and the little toe can easily press a flexible pressure sensing insole, and their pressing action is more flexible and easy to control, and also does not affect the balance state. Since the lesser toe is more flexible than the little toe, and as the touch force generated by its pressing an insole is more obvious, the pressing action of the lesser toe was selected as the feature signal in this paper.

**Figure 12:**
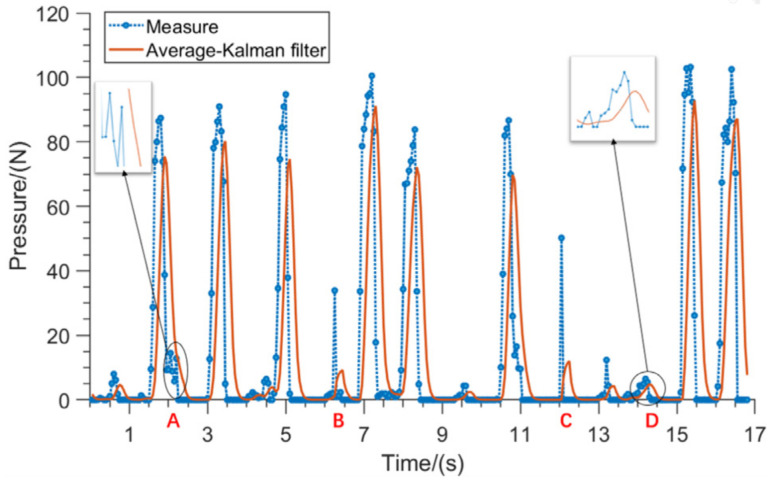
Comparison of the filtering of the contact force signal.

The most flexible big toe pressing action was selected as the characteristic signal, as shown by the blue dotted line in Figure 12, and the lesser toe forms an obvious wave peak after pressing the insole, while the rest of the time the pressure value is lower. Hence, by detecting the wave peak, it can be determined whether there is a pressing action or not. Due to the sensor’s error and external interference, this section used average filtering and Kalman filtering to process the data, and the filtered results are shown as the orange solid line in Figure 12, and it can be seen from points such as A, D, etc., that the signal’s smoothness characteristics were significantly improved; while from points such as B, C, etc., it can be observed that the filtering algorithm had a strong inhibition of random noise, which indicates that this filtering algorithm effectively reduced the noise interference and improved the quality and stability of the signal. The specific processing of this filtering algorithm was as follows:

(1) Recursive averaging filtering of the original tactile signal,

(9) $$x_{k}=\frac{1}{N}\sum_{i=1}^{N-1}y_{k-i}$$

where $y_{k-i}$ is the touch force measurement at moment k−i; $x_{k}$ is the recursive average filter value at moment k; and N is the length of the selection queue.

(2) Kalman filter the obtained recursively averaged filtered signal with the state and measurement Equations, as in Equation (11), where $x_{k-1}$ is the predicted value of the touch force at the moment k−1. $u_{k-1}$ is the system input at moment k−1. $w_{k-1}$ is the process noise at moment k−1. $x_{k}^{-}$ is the estimated value of the touch force at moment k, with the covariance Q. F,B are the system parameters.

(10) $$\left\{\begin{array}{l} x_{k}^{-}=Fx_{k-1}+Bu_{k-1}+w_{k-1}\\ z_{k}=Hx_{k}+v_{k} \end{array}\right.$$

When the lesser toe consciously and actively presses the flexible pressure insole, the system will detect the waveform formed in the tactile force signal curve, and the disabled person will generate additional waveforms to form interference when he/she is in the movement state, but the amplitude of this waveform is obviously smaller compared with that formed by the lesser toe actively pressing the insole, and the interference waveform caused by walking can be shielded by setting the threshold, and at the same time, the threshold can also shield the toe from small mis-touches, which is also effective for the extraction of waveforms from the tactile force curve at rest. It is also effective in extracting the wave peaks of the touch force curve at rest. Therefore, choosing a reasonable threshold value can help distinguish whether the thumb actively makes a pressing action.

Through the above analysis, a pressing action can be identified by recognizing the crests of the tactile force curve and that the pressure is low in the absence of action. Therefore, judgment can be aided by increasing the threshold condition. As each person’s weight and foot shape varies, the exact value of the threshold needs to be adjusted on an individual basis, which is determined by capturing the maximum value of the tactile force signal over some time when walking, standing, or sitting in a normal walking, standing, or sitting position. When the toe presses on the insole, the tactile force timing signal will generate peak signals that exceed the threshold value. Extracting these peak signals that exceed the threshold value can be used to determine whether the toe is consciously performing a pressing action. The specific peak extraction algorithm is as follows, the N-filtered tactile force data are recorded as $a_{1},a_{2},...,a_{N}$, and the specific calculation formula is as follows:

(11) $$\left\{\begin{array}{l} g(x)=\left\{\begin{array}{c} 1,x<0\\ \;0,x\geq 0 \end{array}\right.\\ S=\sum_{i=2}^{N}\frac{g\left(\left(a_{i-1}-t\right)\left(a_{i}-t\right)\right)}{2} \end{array}\right.$$

where g(x) is the negative function defined. $a_{i}$ are the ith filtered touch data. t is the touch threshold. S is the number of peaks above the threshold in the timing curve composed of N data. A set of data points satisfying $g((a_{i-1}-t)(a_{i}-t))=1$ represents a peak signal reflecting a single toe press action. Using the above algorithm, the number of peak signals that satisfy the condition can be obtained, thus identifying whether the toe was pressed or not during that period.

### 4.3. Combined Lidar and Camera Recognition of Object Size and Shape

According to the analysis in Section 2, this system uses a Lidar and monocular camera as the environment sensing module, the camera is used to identify the overall shape of the object, and the Lidar is used to identify the cross-sectional shape and size of the object. In Section 3, we used algorithms such as DTW to identify the cross-sectional shape and size of multiple objects in the environment, and in Section 4, we used the YOLOv8 algorithm to identify the shape of multiple objects in the environment. The system needs to obtain the independent shape and size of each object, so it was necessary to calibrate the objects in the two environments. In this section, a calibration algorithm was designed based on the traditional calibration algorithms of a Lidar camera.

There have been many model studies about camera coordinate transformation, and reference [23] described in detail the principle of camera imaging and the relationship between the camera coordinate system, image coordinate system, pixel coordinate system, and world coordinate system. The transformation relationship between the camera coordinate system and the pixel coordinate system is shown in Equation (12), where $\left(X_{C},Y_{C},Z_{C}\right)$ is a point in the camera coordinate system,$\left(u,v\right)$ are the coordinates of the point corresponding to the pixel coordinate system, f is the focal length of the camera, and $f_{u}$ and $f_{v}$ are the reciprocal of the pixel’s object size in the x,y directions of the image plane, respectively. And $\left(u_{0},v_{0}\right)$ are the origin of the image coordinate system in the coordinates of the pixel coordinate system. The camera coordinate system and the lidar coordinate system are in the same three-dimensional space, they can be converted by Equation (13), where $\left(X_{L},Y_{L},Z_{L}\right)$ is the point corresponding to $\left(X_{C},Y_{C},Z_{C}\right)$ in the Lidar coordinate system, and R,T are the rotation and translation matrices of the two coordinate systems. Combine Equations (12) and (13) and let $Z_{C}A=\left[\begin{array}{ccc} f_{u} & 0 & u_{0}\\ 0 & f_{v} & v_{0}\\ 0 & 0 & 1 \end{array}\right]\left[\begin{array}{ccc} f & 0 & 0\\ 0 & f & 0\\ 0 & 0 & 1 \end{array}\right]$, $R^{\prime }=AR$, $T^{\prime }=AT,$ the conversion equation between the laser coordinate system and the pixel coordinate system can be obtained, as shown in Equation (14), in which $R^{\prime }=\left\lfloor \begin{array}{ccc} r_{11} & r_{12} & r_{13}\\ r_{21} & r_{22} & r_{23}\\ r_{31} & r_{32} & r_{33} \end{array}\right\rfloor$, $T^{\prime }=\left[\begin{array}{c} t_{1}\\ t_{2}\\ t_{3} \end{array}\right]$.

(12) $$Z_{C}\left[\begin{array}{c} u\\ v\\ 1 \end{array}\right]=\left[\begin{array}{ccc} f_{u} & 0 & u_{0}\\ 0 & f_{v} & v_{0}\\ 0 & 0 & 1 \end{array}\right]\left[\begin{array}{ccc} f & 0 & 0\\ 0 & f & 0\\ 0 & 0 & 1 \end{array}\right]\left[\begin{array}{c} X_{C}\\ Y_{C}\\ Z_{C} \end{array}\right]$$

(13) $$\left[\begin{array}{c} X_{C}\\ Y_{C}\\ Z_{C} \end{array}\right]=R\left[\begin{array}{c} X_{L}\\ Y_{L}\\ Z_{L} \end{array}\right]+T$$

(14) $$\left[\begin{array}{c} u\\ v\\ 1 \end{array}\right]=A\left(R\left[\begin{array}{c} X_{L}\\ Y_{L}\\ Z_{L} \end{array}\right]+T\right)=R^{\prime }\left[\begin{array}{c} X_{L}\\ Y_{L}\\ Z_{L} \end{array}\right]+T^{\prime }=\left\lfloor \begin{array}{ccc} r_{11} & r_{12} & r_{13}\\ r_{21} & r_{22} & r_{23}\\ r_{31} & r_{32} & r_{33} \end{array}\right\rfloor \left[\begin{array}{c} X_{L}\\ Y_{L}\\ Z_{L} \end{array}\right]+\left[\begin{array}{c} t_{1}\\ t_{2}\\ t_{3} \end{array}\right]$$

**Figure 13:**
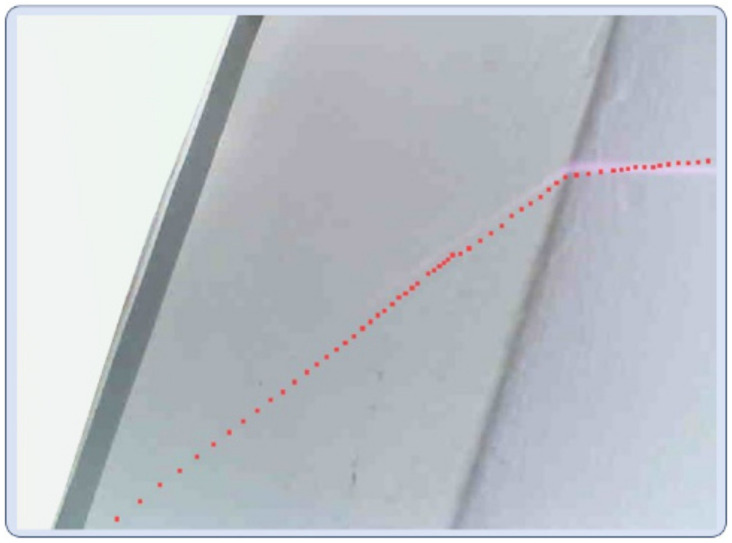
Two-dimensional Lidar–camera calibration algorithm.

The essential task of the calibration of the Lidar and camera is to find $R^{\prime },T^{\prime }$, and this paper used 2D Lidar, and uniformly set its *Z*-axis coordinate to 0. The solution algorithm of this paper was as follows: First of all, 15 groups of points were selected corresponding to $\left(u,v\right)$, $\left(X_{L},Y_{L}\right)$ coordinates, in order to better find the corresponding points, this paper formed the object with a prism to select the marking points, as shown in Figure 13. Therefore, we can obtain the prism in pixel coordinates for the linear Equation au+bv+c=0, *a*, *b*, *c* for the Equation coefficients, and the joint Formula (14) can be the Formula (15), so the solution to finding the $R^{\prime },T^{\prime }$ can be transformed into solving for the values of $r_{11},r_{12},t_{1},r_{21},r_{22},t_{2}$. Then, the collected coordinate data are transformed and brought into Equation (15) to construct a system of transcendental Equations, and $R^{\prime },T^{\prime }$ can be obtained by solving the system of transcendental Equations using the SVD (singular value decomposition) method. As shown in Figure 13, after calibration using the above algorithm, the Lidar points (red points in the figure) were correctly projected onto the image.

(15) $$\left\{\begin{array}{l} a\left(r11x+r12y+t1\right)+b\left(r21x+r22y+t2\right)+c=0\\ \left\lfloor \begin{array}{cc} \begin{array}{ccc} ax & ay & a \end{array} & \begin{array}{ccc} bx & by & b \end{array} \end{array}\right\rfloor \left[\begin{array}{c} \begin{array}{c} r_{11}\\ r_{12}\\ t_{1} \end{array}\\ r_{21}\\ \begin{array}{c} r_{22}\\ t_{2} \end{array} \end{array}\right]=-c \end{array}\right.$$

### 4.4. Integrated Control Methods for Prosthetic Hands

According to the analysis in Section 2, the grasping posture of the prosthetic hand mainly depends on the shape and size of the object, and in this paper, we designed grasping postures for different kinds of objects. In Section 3, the objects were classified as columns, balls, and slices. As shown in Table 2, for columnar objects, if the cross-section size is small, we use the thumb, index finger, and middle finger to pinch and grasp the object. If the size is a little larger, we use five fingers to encircle the object. If the size is larger, we use the thumb and the rest of the fingers to pinch the object. For balls, if the size is small, we use the thumb and index finger to pinch and grasp the object. If the size is large, we use five fingers to try to encircle the object. For slice objects, if the cross-section size is small, we use three fingers to grasp the object in the direction of the cross-section. If the size is large, we use five fingers to pinch and grasp the object in the direction of thickness. For non-regular objects, a default action was designed to grasp the object, i.e., the hand is opened to its maximum extent and then closed.

Based on the above analyses of environment perception and grasping posture, this paper designed a novel control strategy for the prosthetic hand. When there are multiple objects in the environment, the prosthetic hand identifies the object located in the middle, so the subsequent gesture assignment is based on the information of the center object. The core formula of this control method is shown in Equation (16), where P is the result of the operation of the manipulator, ${State}_{plam}$ is the grasping gesture of the prosthetic hand, which is a function of the shape, size, and whether to press or not to identify the object, taking the value of one of the seven kinds of actions plus the default action in Table 2, which are $\left\{s_{1},s_{2},s_{3},s_{4},s_{5},s_{6},s_{7},s_{8}\right\}$. And Pr takes the value of {0,1}, which means no pressing and pressing insole, respectively; if pressing, then this means a need to release the grasping object, ${State}_{plam}$ takes a value $s_{8}$, if no pressing, then this means ready to grasp, according to the shape and size of the object corresponding to $s_{1}-s_{7}$. I is the manipulation intention of the hand-helper system, which takes a value {0,1}, I=0 means manipulate manipulator, I=1 means no intention.

To manipulate the manipulator, there are two ways to activate the manipulation intention of the prosthetic hand in this paper, i.e., the user actively presses the insole (Pr=1) or the system automatically recognizes (Pr=0). If the user presses the insole, I=1, and if the system recognizes automatically, the expression of I is I=μ∗G(X,Y), where μ takes the values of {0,1}, which represent that the upper limb is in the motion or static state, respectively, and G(X,Y) is the function of *X* and *Y*. *X* represents the position of the end of the hand, which takes the values $\left\{Pos_{init},Pos_{front},Pos_{near}\right\}$ and represents that the hand is in the initial state, and G(X,Y) is the function of *X* and *Y*. *X* represents the position of the end of the hand, and the values $\left\{Pos_{init},Pos_{front},Pos_{near}\right\}$, which represent the hand in the initial state, hand in front of the torso, hand near the torso, respectively. *Y* represents the existence of an object in front or not, taking the value of {0,1}, and G(X,Y) takes the value of {0,1}, taking 1 when and only when $X=Pos_{front}$ and Y=1, and 0 otherwise.

(16) $$\left\{\begin{array}{l} P={State}_{plam}\;*\;I\\ {State}_{plam}=\left\{\begin{array}{c} F\left(Size,Category\right)\;,Pr=0\;\\ s8\;,Pr=1 \end{array}\right.\\ I=\left\{\begin{array}{c} \mu \;*\;G\left(X,Y\right)\;,Pr=0\\ 1\;,\;Pr=1 \end{array}\right.\\ G\left(X,Y\right)=\left\{\begin{array}{l} 0\;\;,\;\;\;\;\;\;\;\;\;\;\;\;\;\;\;\;\;\;\;\;\;\;\;\;\;\;\;\;REST\\ H\left(X,Y\right),\;\;\;\;X=Pos_{front}\;and\;Y=1 \end{array}\right. \end{array}\right.$$

## 5. Experiments and Discussion

### 5.1. Shape and Size Recognition of Object Sections

**Figure 14:**
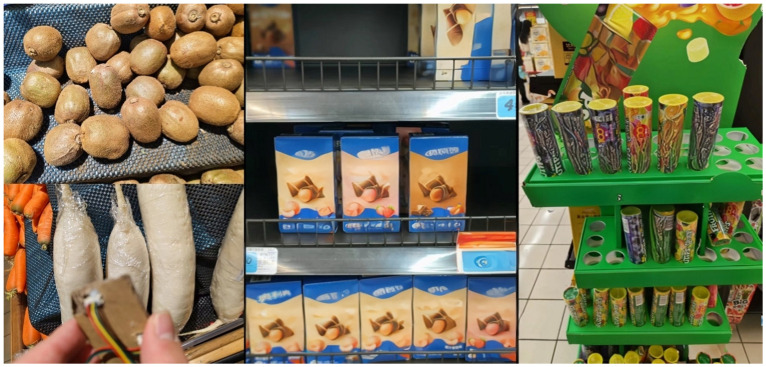
Experimental environment.

In this paper, Lidar is used to obtain the cross-sectional shape and size of objects. To test the recognition effectiveness of the method, this section used Lidar to sense complex environments with multiple objects, as shown in Figure 14. The experimental environment contained common fruit and vegetable stalls and supermarket shelves scenes.

In Section 3.2, cross-sectional shapes of common objects were classified into three categories: circular, straight-line segment, and L-shape. Circular cross-sections are found in objects like cylinders and spheres, straight-line segments in prisms (front view), and L-shapes in prisms (oblique view). Three types of circular cross-section objects (kiwi, radish, snacks, numbered 1–3), three types of straight-line segment objects (orthogonal food boxes, paper towel packets, numbered 4–6), and two types of L-shaped objects (oblique boxes, numbered 7–8) were recognized over 10 trials, with the results summarized in Table 3.

In Table 3, “Object Size” refers to the cross-sectional grasping size, “Successful Positioning” is the number of times the object was located, “Successful Shape Recognition” is the number of times the cross-sectional shape was correctly identified, “Average Recognition Size” is the mean size across 10 trials, “Shape Recognition Rate” is the ratio of successful recognitions, and “Dimensional Tolerance” is the absolute difference between the average recognition size and the actual object size. The results show that the method effectively identified objects of various types and sizes, with the dimensional tolerance within acceptable limits.

### 5.2. Integrated Control Experiment for Prosthetic Hand

To verify the applicability and feasibility of the integrated control method of the prosthetic hand, this experiment used the prosthetic hand to complete upper limb actions. To make the experimental results more reliable and universal, this section designed the experimental actions in Table 4 based on different scenarios and objects of different shapes and sizes.

Drinking water, eating fruit, making a phone call, and shopping were the actions where the system automatically identified the object and assigned a grasping gesture. These actions roughly followed the following process: first, the upper limb moved to the vicinity of the object, the IMU detected the upper limb motion state. Second, the Lidar and the camera located and identified the object, then the prosthetic hand grasped the object according to the corresponding gesture. Finally, the prosthetic hand was released by pressing on the insole. For putting on a hat, the prosthetic hand was actively controlled by the user, with the following process. The process was as follows: The upper limb moved towards the hat, the IMU detected the upper limb motion state, the user took the initiative to press the insole to control the prosthetic hand to grasp the hat and put it on the head, finally pressing the insole to release the prosthetic hand.

**Figure 15:**
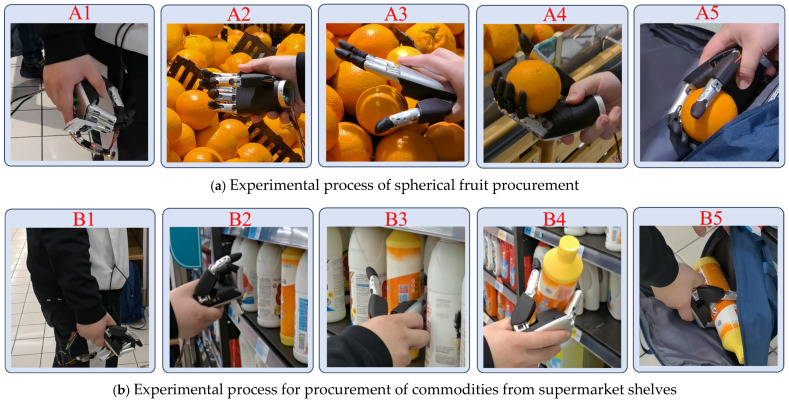

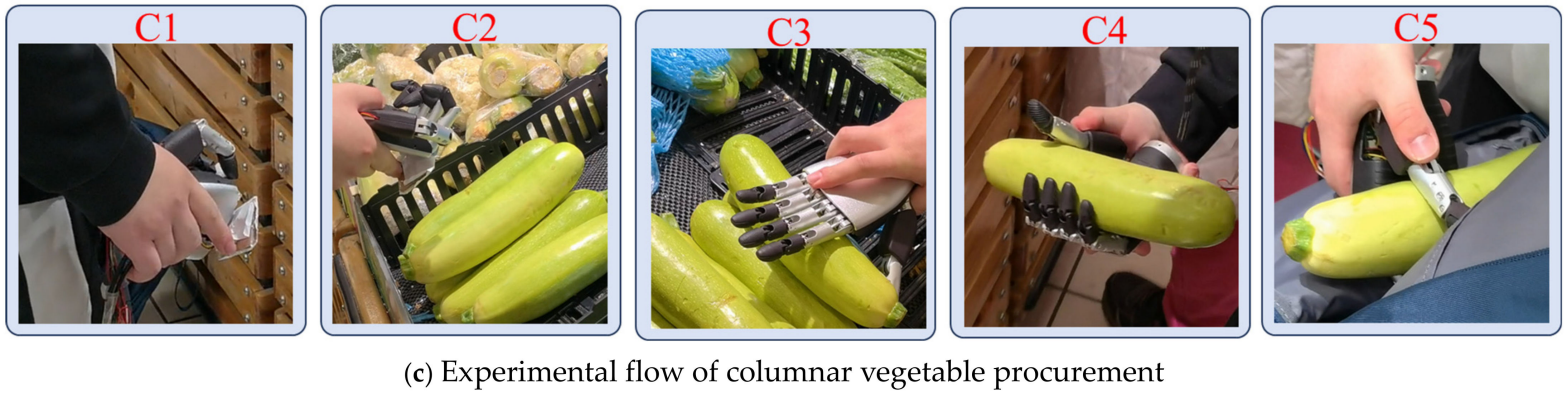
Shopping experiment process.

In the experiment, 10 volunteers were invited to complete the above actions, and each volunteer wore the experimental equipment to repeat each action 20 times using the above action process, after receiving training. The experimental process and data were recorded. The equipment required for the experimental process is shown in Figure 1, where the volunteers wore flexible pressure sensing insoles on the soles of their feet, inertial sensing units on their upper limbs, and robotic hands, as well as a 2D Lidar and a monocular camera, to simulate the use of the environment for people with upper limb disabilities. Then, the experimental process was described in detail, limited by space, focusing on showing the flow of shopping actions as shown in Figure 15. The actions of the shopping process for supermarket fruits were used as an example to analyze the behaviors and data of the experimental process.

**Figure 16:**
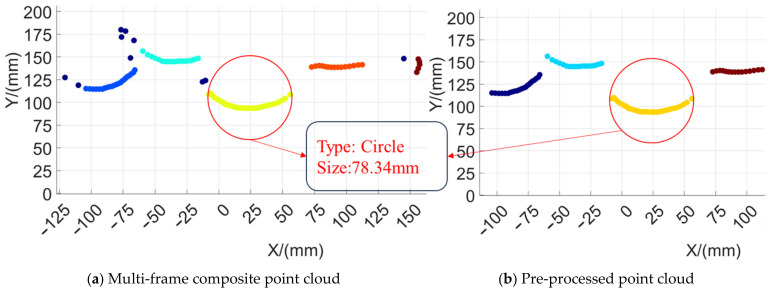
Spherical fruit size recognition results.

**Figure 17:**
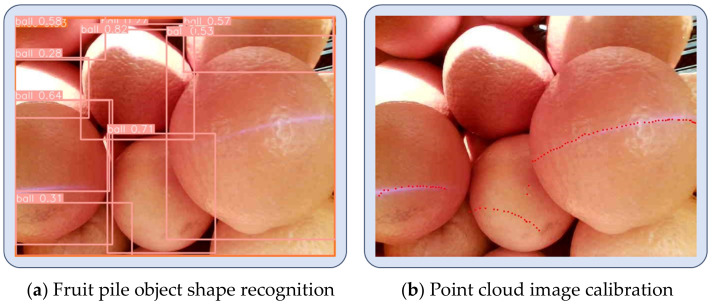
Fruit object recognition and calibration.

Shopping for fruits from a fruit pile is a common scene in daily life, volunteers wearing the prosthetic hand control device completed the action from the initial state to selecting and grasping the object to putting the object into a bag, recording the key steps of the experimental process as well as the data of multiple sensors. The experimental process of spherical fruit purchasing is shown in Figure 15a. The 2D Lidar recognized the point cloud data of the object and the recognition results are shown in Figure 16b. The results of the camera collecting the fruit pile object and recognizing the shape of the object are shown in Figure 17a, and the calibration between the monocular camera and the two-dimensional Lidar is shown in Figure 17b. The user completed the complete fruit object sourcing action, which can be decomposed into five main steps $A_{1}-A_{5}$ as shown in Figure 15a. During the $A_{1}$ time, both the prosthetic hand system and the volunteer were ready and in the initial state, during the $A_{2}$ time, the upper limb drove the robotic hand to gradually move towards the fruit pile, the inertial sensing unit monitored the movement state of the upper limb in real time, and the upper limb was in the stationary state when detected. When the upper limb was in a stationary state and the palm of the hand was located in front of the body, the 2D Lidar started to detect whether there was an object near the palm of the hand, and when an object was detected, the 2D Lidar and the monocular camera were activated to identify the size and shape of the object, respectively, and the point cloud obtained from the scanning of the environmental objects by the 2D Lidar was synthesized from multiple frames, as shown in the above picture in Figure 16. The point cloud was pre-processed with noise reduction and DBSCAN clustering to obtain the four fruits shown in the following picture in Figure 16. The four fruits shown in the following picture are marked by different colors and the target object was located based on the rule that the target object is usually located in the center, i.e., the red circled object shown in Figure 16. The system recognized that the cross-section had a circular shape and its size was 78.34 mm, and the system obtained the calibration result shown in Figure 17b according to the 2D Lidar–monocular camera calibration algorithm. The system obtained the position of the target object in the image through coordinate mapping and used the object shape recognition model trained by YOLOv8 to identify the shape of the target object as a sphere, while the grasping strategy for larger-sized spherical objects was as shown in Table 2, i.e., using five fingers to try to encircle the object. During $A_{3}$ time, the system controlled the manipulator to execute the action according to the assigned grasping strategy. During $A_{4}$ time, the system completed the grasping of the object, and then the upper limb drove the manipulator towards the inside of the shopping bag. At time $A_{5}$, the manipulator placed the fruit inside the shopping bag, and then the system controlled the manipulator to release and return to the initial state, after recognizing the user’s lesser toe pressing down on the flexible pressure-sensing insole. At this point, the complete process of the spherical fruit purchasing action was completed. During the whole process, the inertial sensing unit and the flexible pressure sensing insole accurately identified the gripping timing of the robot, the monocular camera, and the 2D Lidar successfully perceived the environmental information, located the target object, and identified the size and shape of the target object.

The volunteers completed all the actions shown in Table 4, the experimental data and completion of each experiment were recorded, and the experimental results are displayed in Table 5. Here, Num indicates the number of times that the volunteers completed the corresponding actions in total, PRN indicates the number of times that the inertial sensing unit correctly recognized the position of the hand for all the times of the experiments, PRR indicates the ratio of the number of times that the upper limb position was recognized, TON indicated the number of times the Lidar and camera accurately located and identified the object in all the experiments, TOR is the ratio of the target object recognitions to the number of experiments, FPN indicates the number of times the flexible pressure sensing insole successfully identified the lesser toe pressing action, FPR is the ratio of the number of lesser toe pressing recognitions to the number of experiments, ACN indicates the number of times the corresponding action was completed for all times of the experiments, and SR represents the ratio of the number of times the action was completed to the number of experiments, and the average values of each recognition rate and completion rate are also calculated in the table.

The following conclusions can be drawn from the experimental results shown in Table 5.

(1) The position of the upper limb and the recognition of the motion state of the upper limb represented the action intention of the upper limb, and the average accuracy of its recognition was 94.13%, and the recognition rate of the different actions did not differ much, which met the requirements of the different user scenarios. This demonstrates that the method of recognition using the intention of the upper limb in the system has a high degree of feasibility and universality.

(2) Target recognition represents the environment perception ability of the system, which used 2D Lidar to recognize the size of the object and a monocular camera to recognize the shape of the object, and the average value of the target recognition rate was 91.75%, recognizing most of the scenes with good accuracy. The target recognition rate was similar for different scenes, indicating that the object recognition algorithm of the present system has good usability and generality.

(3) The recognition of the lesser toe pressing action represents the system’s ability to recognize the user’s active human–computer interaction intention. First, it had a high success rate for drinking water, eating fruits, making phone calls, shopping, and other scenarios, with an average recognition rate as high as 98.88%. Second, it also had a recognition rate as high as 98% in the hat wearing scenario, which relied on active human–computer interaction to control the prosthetic hand, which indicates that the user’s lesser toe pressing on the flexible pressure sensing insole was capable of controlling the prosthetic hand system efficiently. This indicates that the user could control the prosthetic hand system efficiently by pressing the flexible pressure-sensing insole with his/her lesser toe.

(4) The success rate represents the rate at which each step in the whole action process was completed, and the average success rate was 91.63% for the scenario of automatic recognition and sensing by the system. The success rate was similar in the different scenarios, and the recognition rate was 98% for the scenario that relied on active human-computer interaction only, which demonstrates that the system designed in this paper has a high degree of feasibility and universal applicability.

(5) Due to the close cooperation of many sensors in the scene of automatic recognition and perception, the final success rate was not higher than the lowest success rate in each link, and at the same time, the target recognition was affected by the variety of complex environments, and the recognition rate was generally the lowest success rate in each link, which could be improved by adapting more point cloud scenes and increasing the number of picture training sets. This will be improved in future research.

## 6. Conclusions

In this study, we proposed a new method for recognizing the intention of upper limb movements: detecting the scale information of a grasped object based on the visual fusion of information from a miniature Lidar and pinhole camera, so as to automatically control the grasping posture of the manipulator; controlling the flow of grasping movements based on arm motion and toe haptic information. The hardware device in this paper is suitable for integrated mounting on the finger and also does not need to be in contact with the human skin, a feature that provides a great improvement in comfort and aesthetics compared to myoelectric- and electroencephalographic-controlled manipulators; the grasp and release commands of the manipulator used a human-in-the-loop decision-making method, which provides better interference immunity and reliability compared to manipulators controlled by signals from myoelectrics, EEGs, and speech. In our future research, we plan to more finely classify object shapes and grasping postures, and further delve into the stability and smoothness of the grasping behavior of the prosthetic hand. In addition, in the course of our research, we found that the comfort of the prosthetic hand was very important, all the current robotic hand for upper limb disabled people must be installed on the disabled person’s residual limb skin through the prosthetic limb, which will cause discomfort such as sweating, skin inflammation, and will also cause pain in the skin due to the friction, and the implantable prosthesis of the upper limb may be a fundamental way to solve this problem.

## Figures and Tables

**Table 1:** Training hardware environment.

CPU	Graphics Card	Memory	Video Storage
11th Gen Intel(R) Core(TM) i7-11700k	NVIDIA GeForce RTX 3080	32 GB	10 GB

**Table 2:** Object grasping posture design.

Cross-Section Size	0–20 mm	20–50 mm	50–150mm
**Column**	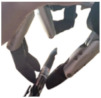	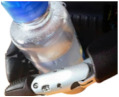	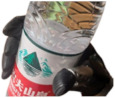
**Diameter**	**0–50 mm**	**50–150 mm**	
**Ball**	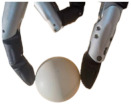	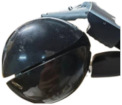	
**Cross-section size**	**0–50 mm**	**50–150 mm**	
**Slice**	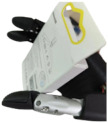	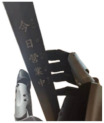	

**Table 3:** Recognition results of eight kinds of object cross-sections.

Object Type	Object No.	Object Size (mm)	Positioning Successes	Number of Successful Shape Recognition	Average Recognized Size (mm)	Shape Recognition Rate	Dimension Tolerance (mm)
**Circular**	1	43	10	10	46	100%	3
	2	71	10	10	76	100%	5
	3	108	10	9	119	90%	11
**Straight-line**	4	11	10	10	12	100%	1
	5	34	10	10	36	100%	2
	6	71	10	10	73	100%	2
**L-shape**	7	70	10	9	63	90%	7
	8	95	10	9	84	90%	11
**Average**						96%	5

**Table 4:** Experimental actions and action flow.

Action	Action Flow
**Drink water**	Upper limb moves towards the cup→recognizes the cup→grasps and moves to the mouth→retracts and releases
**Fruit-eating**	Move the upper limb towards the fruit stand→select and recognize a fruit→grasp and move to the mouth→release
**Call**	Upper limb moves towards the mobile phone→recognizes the mobile phone→grasps and moves to the ear→retracts and releases it
**Shop**	Upper limb moves towards the shelf→selects and recognizes the product→grasps and moves it to the shopping cart→releases it
**Wear a hat**	Upper limb moves to near the hat→toes press the insole→grasps the hat and moves it to the head→releases it

**Table 5:** Experimental results of the integrated control of the prosthetic hand.

Action	Num	PRN	PRR	TON	TOR	FPN	FPR	ACN	SR
**Drink water**	200	189	94.50%	185	92.50%	198	99.00%	185	92.50%
**Fruit-eating**	200	188	94.00%	182	91.00%	199	99.50%	181	90.50%
**Call**	200	190	95.00%	184	92.00%	197	98.50%	184	92.00%
**Shop**	200	186	93.00%	183	91.50%	197	98.50%	183	91.50%
**Average**			94.13%		91.75%		98.88%		91.63%
**Wear a hat**	200					198		196	98.00%

## Data Availability

The data presented in this study are available on request from the corresponding author.
